# The relation between celiac disease, nonceliac gluten sensitivity and irritable bowel syndrome

**DOI:** 10.1186/s12937-015-0080-6

**Published:** 2015-09-07

**Authors:** Magdy El-Salhy, Jan Gunnar Hatlebakk, Odd Helge Gilja, Trygve Hausken

**Affiliations:** 1 Section for Gastroenterology, Department of Medicine, Stord Hospital, Stord, Norway; 2 Section for Neuroendocrine Gastroenterology, Division of Gastroenterology, Department of Clinical Medicine, University of Bergen, Bergen, Norway; 3 National Centre for Functional Gastrointestinal Disorders, Department of Medicine, Haukeland University Hospital, Bergen, Norway; 4 National Centre for Ultrasound in Gastroenterology, Department of Medicine, Haukeland University Hospital, Bergen, Norway

## Abstract

Wheat products make a substantial contribution to the dietary intake of many people worldwide. Despite the many beneficial aspects of consuming wheat products, it is also responsible for several diseases such as celiac disease (CD), wheat allergy, and nonceliac gluten sensitivity (NCGS). CD and irritable bowel syndrome (IBS) patients have similar gastrointestinal symptoms, which can result in CD patients being misdiagnosed as having IBS. Therefore, CD should be excluded in IBS patients. A considerable proportion of CD patients suffer from IBS symptoms despite adherence to a gluten-free diet (GFD). The inflammation caused by gluten intake may not completely subside in some CD patients. It is not clear that gluten triggers the symptoms in NCGS, but there is compelling evidence that carbohydrates (fructans and galactans) in wheat does. It is likely that NCGS patients are a group of self-diagnosed IBS patients who self-treat by adhering to a GFD.

## Introduction

The three most important food grains worldwide are wheat, maize, and rice [1]. However, the ability to produce high yields of wheat under a broad range of conditions, so that it can be cultivated successfully at global latitudes from 67° N in Scandinavia to 45° S in Argentina, renders it particularly useful as a food source [1]. The nutritional needs following the two world wars and the exponential growth of the world population resulted in the global production and consumption of wheat expanding hugely by the end of the twentieth century [1–3]. Moreover, several countries in Asia have reduced their consumption of rice considerably during the last years, probably in favor of wheat consumption [4] [Hossain M. Food and Agriculture Organization of the United Nation. 2004. www.fao.org/rice2004/en/e-001.htm] (Table 1). The high consumption of wheat is not only attributed to its adaptability and potential for high yields, but also to its viscoelasticity, which allows it to be processed into several food items such as bread, baked products, and pastas [1]. Wheat and wheat-based products make substantial contributions to the dietary intake of protein, dietary fiber, minerals (especially iron, zinc, and selenium), vitamins, phytochemicals, and energy [1]. It has been estimated that several billion people rely on wheat for a substantial part of their diet [1].

**Table 1:** The consumption of rice in some Asian countries kg/person/year

Time period	Countries			
	Japan	Thailand	South Korea	North Korea
1970–72	89	152	119	82
1999–2001	59	109	88	78

Data from [Hossain M. Food and Agriculture Organization of the United Nation. 2004. www.fao.org/rice2004/en/e-001.htm]

White flour comprises about 80 % starch and 10 % protein [1, 5]. The indigestible oligosaccharides such as fructo-oligosaccharides and fructans constitute 13.4 % of the dietary fiber in wheat [5, 6]. Moreover, wheat contains a considerable amount of the indigestible oligosaccharides galactans [6, 7]. Gluten constitutes 80 % of the wheat proteins, and comprises two major groups: the glutenins and the gliadins (prolines) [8, 9]. The glutenins occur in two forms, the high- and low-molecular-weight fractions [8–11], while the gliadins exist as three structural forms, α-, ω-, and γ-gliadins [8–11]. Glutenins and gliadins undergo partial digestion in the upper gastrointestinal tract, resulting in the formation of various native peptide derivatives that are resistant to digestion by the gastrointestinal proteases [12].

Despite the many beneficial aspects of consuming wheat products, it can cause several diseases such as celiac disease (CD), wheat allergy (WA), and nonceliac gluten sensitivity (NCGS) [12, 13]. The prevalence rates of CD, WA, and NCGS are estimated to be 0.5–2 % in the Western population [14–23], 0.2–0.5 % in the European population [24], and 0.55–6 % of the USA population [11, 25], respectively. CD is an immunological response to ingested gluten that results in small-intestine villous atrophy with increased intestinal permeability and malabsorption of nutrients [26]; WA is characterized by an IgE-mediated response against various wheat components that results in respiratory or gastrointestinal symptoms [27]; and NCGS is characterized by both gastrointestinal and extragastrointestinal symptoms that are triggered by the ingestion of wheat products (possibly due to the gluten content). These symptoms improve after removing wheat products from the diet, and relapse following a wheat challenge. The gastrointestinal symptoms in NCGS are abdominal pain, diarrhea or constipation, nausea, and vomiting, and the extragastrointestinal symptoms are headache, musculoskeletal pain, brain fog, fatigue, and depression [28, 29]. NCGS is often perceived by the patients themselves, leading to self-diagnosis and self-treatment.

Patients with irritable bowel syndrome (IBS), CD, and the recently debated diagnosis of NCGS exhibit similar gastrointestinal and extragastrointestinal symptoms [4, 30–37]. Application of symptom-based diagnosis criteria such as the Rome III criteria could result in diagnosing patients with CD or NCGS as having IBS. However, it has been suggested that these three conditions overlap [4, 12]. The aim of this review was to elucidate this possible overlapping of these three conditions and to find out a way to separate them in everyday clinical practice.

## IBS and CD

### The connection between IBS and CD: is it a misdiagnosis or an overlap?

As mentioned above, there is overlap in the symptoms of IBS and CD. Since the diagnosis of IBS is based mainly on symptom assessment using symptom criteria such as the Rome III criteria, there is a risk of CD patients being misdiagnosed as having IBS. The situation is complicated even further by the fact that the abdominal symptoms in both IBS and CD patients are triggered by the ingestion of wheat products. However, whereas this is caused by gluten allergy in CD patients, it is attributed in IBS patients to the long-sugar-polymer fructans in wheat [38]. The prevalence of CD patients among IBS patients who have been misdiagnosed using symptom criteria for IBS has varied between studies from 0 to 31.8 %, but in most studies it seems to lie in the range 0.4–4.7 % [14, 23, 39–48]. Regardless of this variation in the prevalence of CD patients who have been misdiagnosed with IBS between studies, it is a considerable number that should not be disregarded. These findings have led to the British Society of Gastroenterology recommending that CD should be excluded in all patients referred with IBS [49], and the American College of Gastroenterology have advised the exclusion of CD in patients with diarrhea-predominant IBS and IBS with a mixed bowel pattern [47]. Based on the international guidelines, published data, and our own clinical experience, we believe that all referred IBS patients should be tested for CD with serologic testing, including a combination of tissue transglutaminase (tTG) and deamidated gliadin peptide (DGP) antibodies, which have demonstrated a high sensitivity and specificity [50], and when there is doubt, duodenal biopsy samples should be taken. In our clinical experience, it is not uncommon for patients to test positive for tTG but negative for DGP, and vice versa, making it necessary to perform both tests in patients. This suggestion is supported by reports that the time between CD symptom onset and correct medical diagnosis is between 5.1 and 11.7 years [51, 52]. This period is prolonged from an average of 7 years in CD patients with no prodromal IBS to 10 years in those with prodromal IBS [53].

The reported prevalence of positivity to antigliadin antibodies (AGA; IgG and IgA) without any confirmation of positivity to tTG or DGP antibodies in the blood of IBS patients has varied from 5 to 17 % [14, 44, 54] to as high as about 50 % [55, 56]. AGA positivity has been reported to have a good sensitivity, but a low specificity for CD [57], and the serum of 12–15 % of healthy subjects is positive for AGA [14, 44, 57, 58]. This raises an interesting question: why would a healthy subject have these antibodies to gliadin with no clinical implications? One could speculate that mucosal damage and failure of the mucosal barrier caused by acute or chronic alcohol consumption [59], or by a bout of gastroenteritis, would allow the immunogenic peptides resulting from the partial digestion of glutamines and gliadins to enter the lamina propria, where they could interact with immune cells, resulting in the production of AGA. Acute and chronic alcohol intake as well as gastroenteritis are not uncommon, and so this could explain both the high prevalence of AGA positivity in healthy subjects and the lack of clinical relevance of AGAs in both the healthy subjects and IBS patients.

### Small-intestine endocrine cells in IBS and CD

**Fig. 1:**
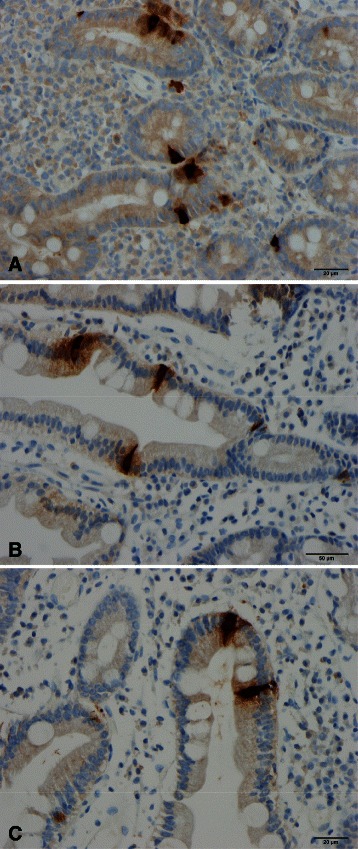
Secretin cells in the duodenum of (**a**) a healthy subject, (**b**) a patient with celiac disease (CD), and (**c**) a patient with irritable bowel syndrome (IBS)

**Fig. 2:**
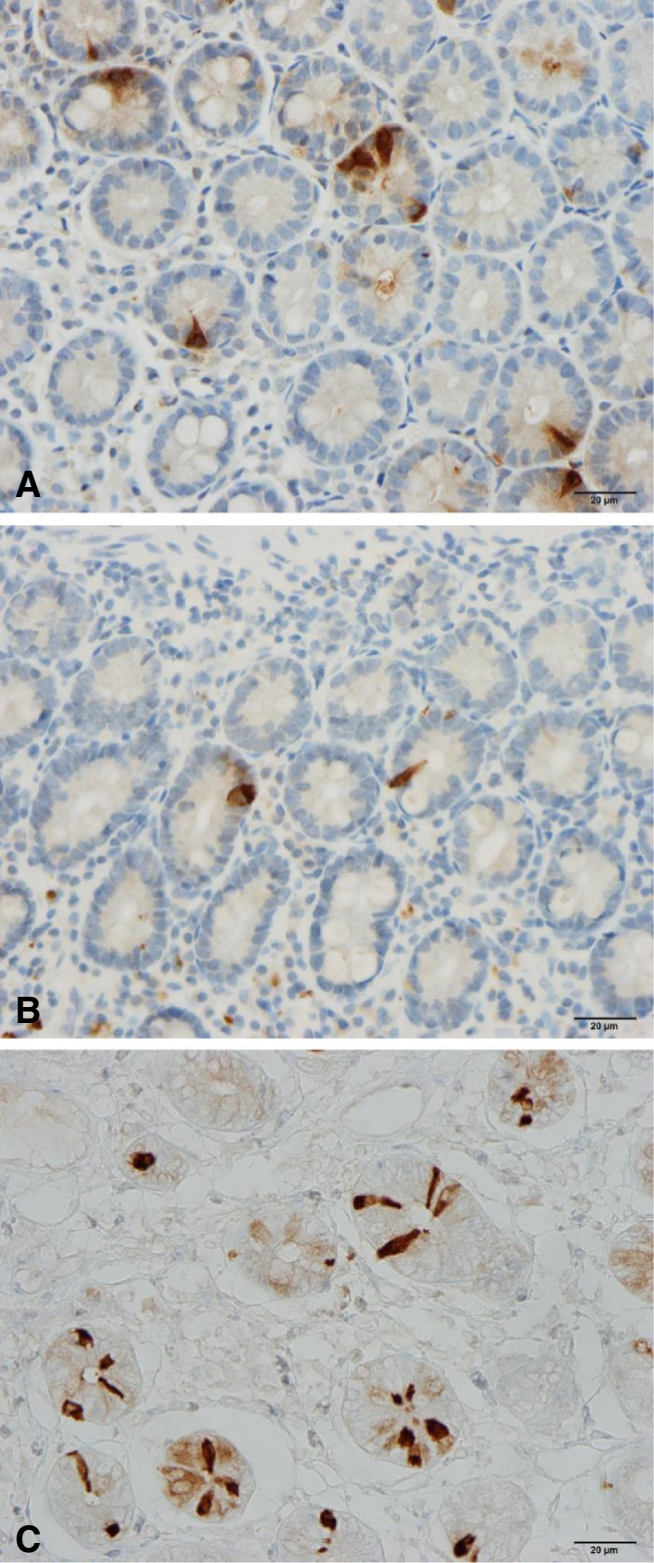
Duodenal cholecystokinin cells in (**a**) a healthy subject, a patient with CD (**b**), and (**c**) a patient with IBS

**Fig. 3:**
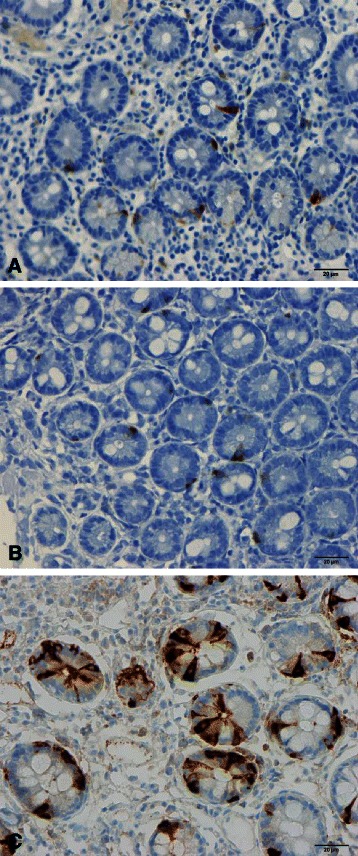
Gastric inhibitory polypeptide (GIP)-immunoreactive cells in (**a**) a healthy subject, (**b**) a patient with CD, and (**c**) a patient with IBS

Abnormalities in the densities of the various types of endocrine cells in the small intestine have been reported in both IBS and CD (Table 2 and Figs. 1, 2 and 3) [60–78], and these abnormalities are considered to play an important role in the symptom development in both of these diseases [63, 79–81]. The pattern of changes in the densities of small-intestine endocrine cells in patients with CD is quite different from that in IBS and postinfectious IBS (PI-IBS).

**Table 2:** Patterns of changes in the densities of small-intestinal endocrine cells in irritable bowel syndrome (IBS), celiac disease, and postinfectious IBS (PI-IBS)

Endocrine cell type	IBS	Celiac disease	PI-IBS
Secretin	Decreased	Decreased	Unknown
Cholecystokinin (CCK)	Decreased	Increased	Increased
Gastric inhibitory peptide (GIP)	Decreased	Increased	Unknown
Serotonin	Unchanged	Increased	Unchanged
Somatostatin	Decreased	Increased	Unknown

### Patients with both CD and IBS

It is not uncommon for patients with CD who consume a gluten-free diet (GFD) and suffer from IBS symptoms to present at the clinic. Reportedly 20–23.3 % of treated CD patients fulfill the symptom-based Rome criteria for IBS [82, 83]. A meta-analysis found that the pooled prevalence of IBS symptoms in patients with treated CD was 38 % [84]. Despite adhering to a GFD, patients with CD who exhibit IBS symptoms have a reduced quality of life as compared with those who do not [82, 83, 85]. It is possible that CD and IBS coexist in some patients [86]; however, it is more likely that the inflammation in CD does not subside completely in some patients after implementation of a GFD, and a low-grade inflammation [82] similar to that seen in PI-IBS may exist [36]. This assumption is supported by certain observations in other inflammatory bowel diseases such as ulcerative colitis and Crohn’s disease, whereby 33–46 % of those with ulcerative colitis and 42–60 % of those with Crohn’s disease exhibit IBS symptoms during remission periods [87–91]. Fecal calprotectin is significantly elevated in ulcerative colitis and Crohn’s disease patients with IBS in remission, compared to those without IBS-type symptoms, indicating the presence of occult inflammation [90].

### IBS and NCGS

#### NCGS

NCGS receives widespread interest from the general public and mass media, and is often confused with the popular assumptions and speculations that the high carbohydrate content of wheat is responsible for negative health aspects such as obesity [92]. The situation is exacerbated by celebrities propagating these speculations as a means of losing weight. The concept of NCGS was first introduced in 1978 with a case report of a patient with abdominal pain and diarrhea who exhibited no abnormalities on small-intestine biopsy samples, whose symptoms improved when they changed to a GFD [93]. A study of eight adult females with abdominal pain, diarrhea, and small-intestine biopsy findings with no significant changes published in 1980 found that symptoms were relieved when the patients adhered to a GFD, and returned after a gluten challenge [94]. Similar results have been reported in patients with nonceliac IBS-like symptoms [95–97]. The withdrawal of wheat products was found to improve these symptoms in double-blind randomized, placebo-controlled studies involving patients with IBS-like symptoms [98, 99].

Whereas some studies involving experimental animals and humans have revealed that exposure to gluten induces intestinal low-grade inflammation, proliferation of peripheral blood monocytes, and enhancement of cytokine production [96, 100–102], others were unable to find any gluten-induced inflammation in NCGS [103]. Similar discrepancies have been reported regarding small-intestine permeability [96, 99, 104].

#### Is gluten responsible for NCGS?

As pointed out by Nijeboer et al. [105], in studies showing an effect on symptoms in NCGS patients, that effect was actually attributable to withdrawal of wheat rather than gluten [96, 98, 99]. A placebo-controlled, crossover study involving patients with IBS-like symptoms who were on a self-imposed GFD [106] found that the gastrointestinal symptoms consistently and significantly improved during a reduced intake of fermentable oligosaccharides, disaccharides, monosaccharides, and polyols (FODMAPs), and these symptoms were not worsened by either a low- or high-dose challenge with gluten. Moreover, in a study involving adults who believed that they had NCGS, 24 % had uncontrolled symptoms despite consuming a GFD, 27 % did not strictly follow a GFD, and 65 % avoided other foods that contained high levels of FODMAPs as additional symptom triggers [107]. The findings of this study lend further support to the notion that the gluten in wheat is not the trigger of NCGS symptoms. However, in a recent randomized, double-blind, placebo-controlled, cross-over study on adults without CD or WA who believe that they suffer from NCGS showed that intake of small amounts of gluten increase the intestinal and extraintestinal symptoms significantly [108].

Together these results indicate that it is not clear that gluten is responsible for triggering NCGS symptoms; instead, it appears that it is the carbohydrate content (fructans and galactans) in the wheat that triggers these symptoms. Indigestible and poorly absorbed short-chain carbohydrates with chains containing up to ten sugars (which are collectively called FODMAPs) occur in a wide range of foods, including wheat, rye, vegetables, fruits, and legumes [109]. These carbohydrates exert osmotic effects in the large intestinal lumen, resulting in an increased water content. They are also fermented rapidly by intestinal bacteria, with consequent gas production [109–111]. Several studies, including some randomized, placebo-controlled studies, have shown that FODMAPS trigger gastrointestinal symptoms in IBS, and that a low-FODMAPs diet reduces symptom severity and improves the patient’s quality of life [112–119].

It is noteworthy that initiation of GFD without dietetic supervision or education can cause inadequacies of nutrient intake including fiber, thiamin, folate, vitamin A, magnesium, iron, and calcium [120]. Furthermore, it is difficult [121] and more expensive to follow a GFD.

#### Connection between IBS and NCGS

The definition of NCGS [28] coincides with that of IBS: they have the same gastrointestinal and extragastrointestinal symptoms. A point of difference may be that NCGS patients’ symptoms improve on withdrawal of gluten and return with gluten ingestion. However, it is not clear that gluten triggers the symptoms in NCGS patients; rather, there is compelling evidence that they are triggered by the fructan and galactan carbohydrate components, which are FODMAPs [106]. Furthermore, a considerable number of patients with NCGS experience no improvement of symptoms despite consuming a GFD, and a large number appear to avoid other food items that contain high levels of FODMAPs in addition to consuming a GFD [107]. It is possible that the frequency of IgG/IgA AGA is higher and the association with human leukocyte antigens (HLA) DQ2 and DQ8 is stronger in these patients [98]. As mentioned above, confirmation of AGA positivity is not a specific test, since a considerable number of healthy individuals are positive for this antibody and HLA DQ2 and DQ8 are common in healthy population.

The fructan contents in gluten-free bread (mostly made of rice/corn), bread made from white wheat flour, and bread made from spelt flour are 0.19 g/100 g, 0.68 g/100 g [6], and 0.14 g/100 g, respectively. In addition, spelt flour contains 16 % less protein (mostly gluten) compared to wheat [122]. Given the likelihood that it is the carbohydrate components of wheat that trigger symptoms in NCGS, spelt products would be a better alternative to wheat than a GFD, which is widely used by IBS patients [113, 114].

The following two statements from the research groups of Murray and Sanders should be emphasized in the ongoing debate regarding NCGS:

“Symptoms, symptom complexes, and symptom characteristics are rarely, if ever diagnostic” [100]

and

“We believe that further work is required in what we perceive as the research fertile crescent of gluten sensitivity! Only then can we better inform practicing clinicians on how to manage this group of patients” [86].

## Conclusions

CD patients experience gastrointestinal symptoms similar to those seen in IBS patients, and are thus at risk of being misdiagnosed as having IBS. Therefore, CD should be excluded in IBS patients, regardless of the subtype. A considerable proportion (20–37 %) of CD patients suffer from IBS symptoms despite adherence to a GFD. It is likely that the inflammation caused by gluten intake does not completely subside in some CD patients, similar to what is seen in inflammatory bowel diseases, whereby a considerable number of patients exhibit IBS symptoms in the remission phase.

It is not clear that gluten triggers the symptoms in NCGS, but there is compelling evidence that carbohydrates (fructans and galactans) in the wheat does. Patients with NCGS exhibit the same gastrointestinal and extragastrointestinal symptoms as those with IBS. Withdrawal of wheat products reduces the symptom severity and improves the quality of life in both NCGS and IBS patients. Furthermore, there are no specific blood tests or radiological or endoscopic examinations that are diagnostic for either NCGS or IBS. It is likely that NCGS patients constitute a group of IBS patients who are self-diagnosed and have self-treated by adhering to a GFD.
